# Primary Stromal Breast Sarcoma with Concomitant Contralateral Carcinoma: A Rare Case from Syria

**DOI:** 10.1155/2019/6460847

**Published:** 2019-09-10

**Authors:** Rawan Al khudari, Mohannad Homsi, Hasan Al zohaily, Maher S. Saifo

**Affiliations:** ^1^Faculty of Medicine, Damascus University, Damascus, Syria; ^2^Department of Oncology, AL-Bairouni University Hospital, Damascus University, Damascus, Syria

## Abstract

Bilateral breast cancers are rare cases encountered and are usually the same type in both sides. Only very few cases were reported to have different histological types of neoplasia involving sarcoma. Moreover, sarcomas rarely originate from the breast as a primary lesion whereas the common presentation is having angiosarcoma following radiotherapy. In this report, we present a rare case of a Syrian 43-year-old woman having two distinct primary lesions in the breasts: invasive ductal carcinoma and contralateral stromal sarcoma.

## 1. Introduction

A breast stromal sarcoma is any tumor originating from the intralobular stroma [1]. They were firstly defined in 1962 by Berg et al. as a “group of mesenchymal malignant tumors with fibrous, myxoid and adipose components excluding malignant cystosarcoma phylloides, lymphomas, and angiosarcomas” [2].

Primary malignant mesenchymal breast tumors (primary breast sarcomas) are uncommon entities that represent 0.2–1.0% of all breast malignancies [3]. Regarding that, bilateral breast cancer is rare and only 2 to 11% of women diagnosed with breast cancer will develop contralateral breast cancer in their lifetime, [4] presenting with two different types of cancers, one of which is stromal sarcoma, which is extremely rare.

In this article, we report a rare case from Syria presenting with bilateral breast cancer, invasive ductal carcinoma, and primary stromal sarcoma in the other side.

## 2. Case Report

A 43-year-old white woman presented with a palpable lump in the right breast to AL-Bairouni University Hospital in September 2016.

She is married with six children, no menstrual disturbances, no history of breast trauma, no exposure to radiation, or no family history of breast cancer.

Clinical examination showed a 3 cm in diameter lump with irregular borders in the superior lateral quadrant of the right breast, with no swollen nodes.

A mammogram of the right breast showed a density in the superior lateral quadrant; the density corresponds with Breast Imaging Reporting and Data System (BIRADS) 4 (suspicious abnormality) [5] which required an excisional biopsy to exclude malignancy.

**Figure 1:**
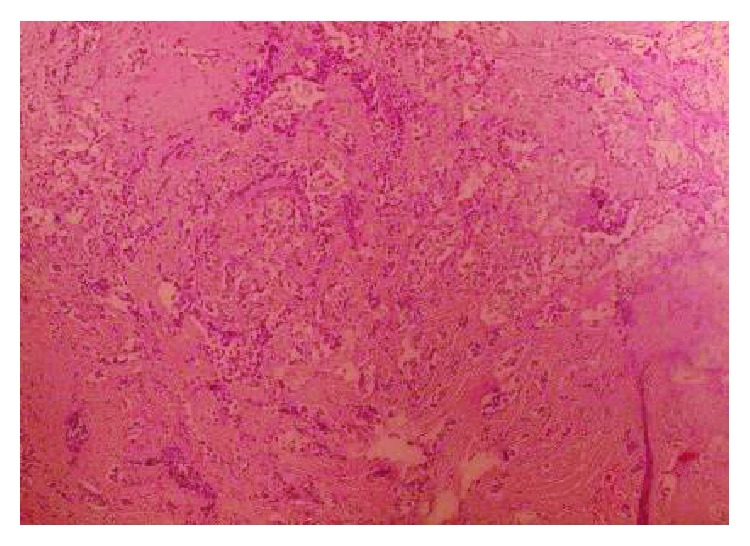
Invasive ductal carcinoma (H&E).

**Figure 2:**
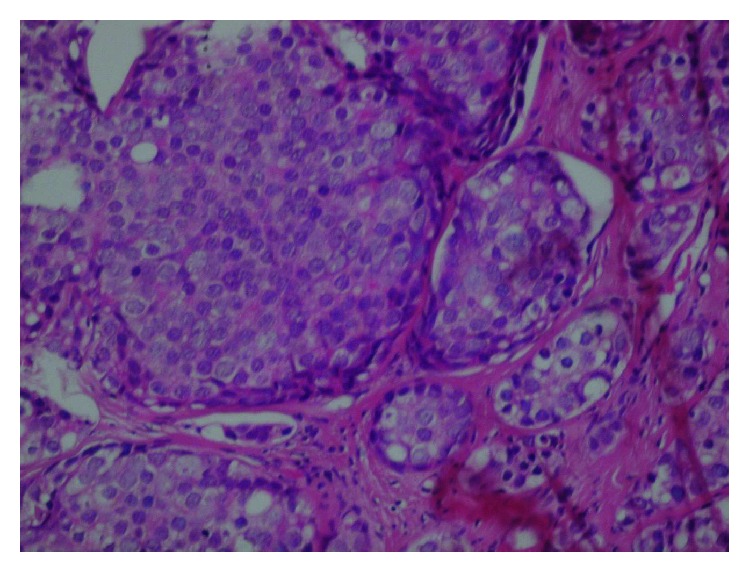
Magnified image showing ductal carcinoma in situ.

**Figure 3:**
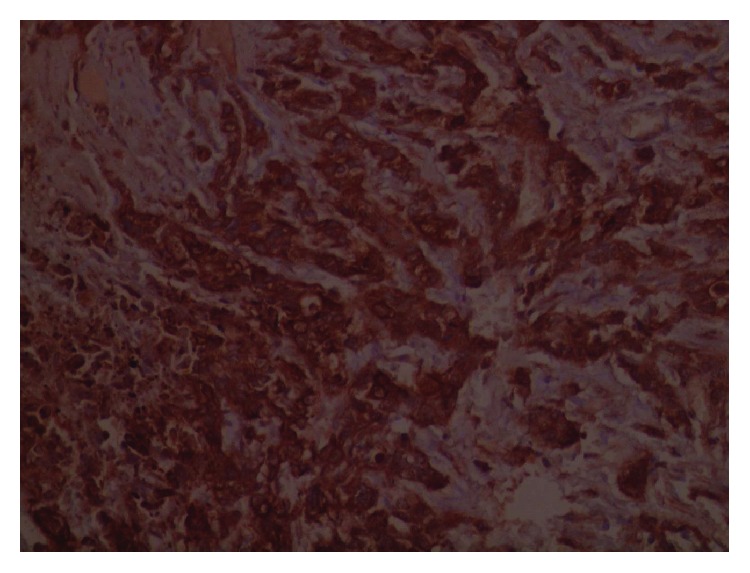
CK7 staining with positive results indicating carcinoma.

The lesion in the excisional biopsy was about 4.5 cm (T_2_). The histopathology exam showed proliferation of epithelial cells of the mammary canals infiltrating to the space between the canals (Figure 1) and in addition to simultaneous ductal carcinoma in situ (Figure 2). CK7 staining proved carcinoma origin (Figure 3). These findings revealed invasive ductal carcinoma grade III. A hormonal receptor test was 10% positive for progesterone receptors and 10% positive for estrogen receptors; a HER-2 test was negative. Staging depending on chest X-ray, bone scintigraphy, and thoracoabdominal CT scan revealing no signs of metastatic disease suggested a T_2_N_0_M_0_ score and a stage IIA tumor.

The patient received neoadjuvant chemotherapy for three months consisting four cycles of EC (epirubicin-cyclophosphamide) according to the National Comprehensive Cancer Network NCCN guideline [6]. Quadrantectomy was planned after the fourth dose. Unfortunately, due to issues related to the war in Syria, there was no possible connection with the patient for about eight months which corrupted her treatment plan.

Later, in August 2017, 8 months after receiving the last dose of chemotherapy, 11 months after the first presentation and mammogram, she presented with two lumps, one for each breast. Physical examination showed palpable lesions; the left breast lump was 3 cm in diameter in the superior lateral quadrant of the breast; with a mild nipple retraction without any discharge, the skin was normal. While the right breast lump was 2 cm in diameter in the superior lateral quadrant of the breast, there was no nipple retraction of the right breast and no swollen axillary lymph nodes on both sides. Differential diagnosis included the recurrence of the primary tumor. We repeated the imaging and histopathology study for confirmation.

A mammogram showed on the right breast an asymmetric density in the upper outer quadrant that falls into the BIRADS 3 (probably benign) category suggesting recurrence. There was neither nipple retraction nor calcification.

From the left breast mammogram, a heterogeneous density was noted in the upper outer quadrant. This had poorly defined margins (speculated) and appeared highly infiltrative. Also, there was a small density with poorly defined margins in the central part, which corresponds to the BIRADS 4 category (suspicious abnormality) with thickened skin and mild nipple retraction, but no calcification, which prompted excisional biopsy from the left breast.

**Figure 4:**
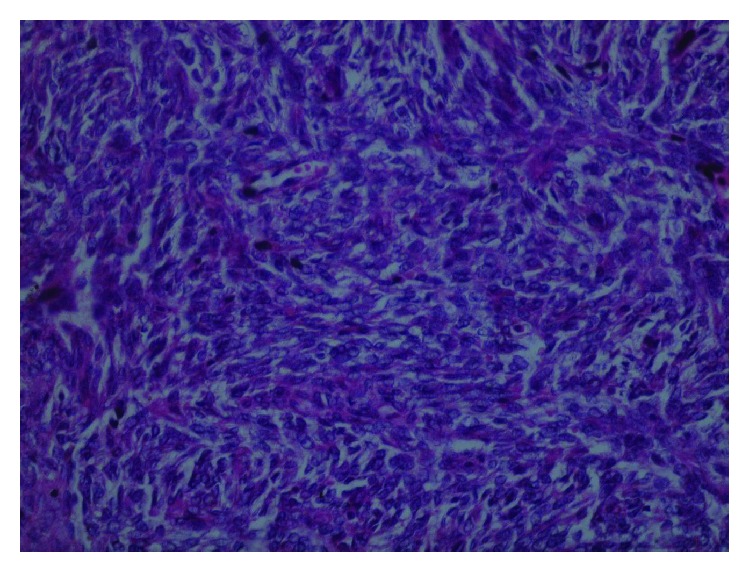
H&E microscopic image of the left mass showing spindle-shaped cells.

**Figure 5:**
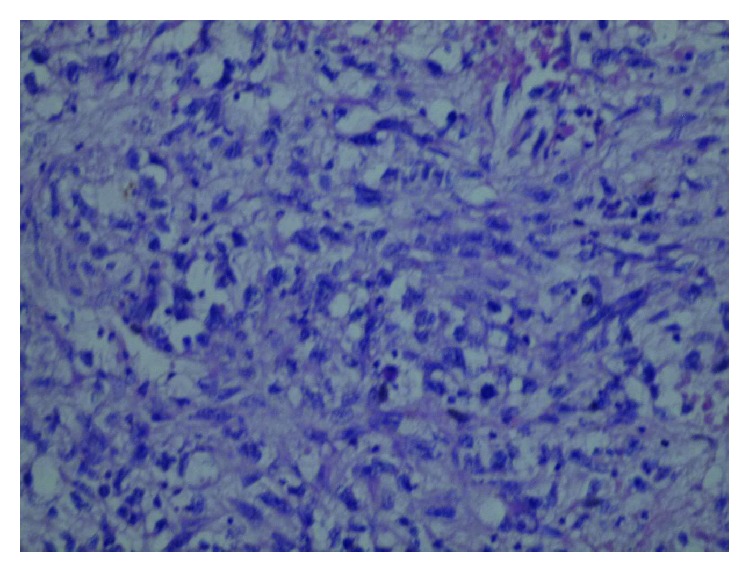
Negative CK7 stain of the left mass.

**Figure 6:**
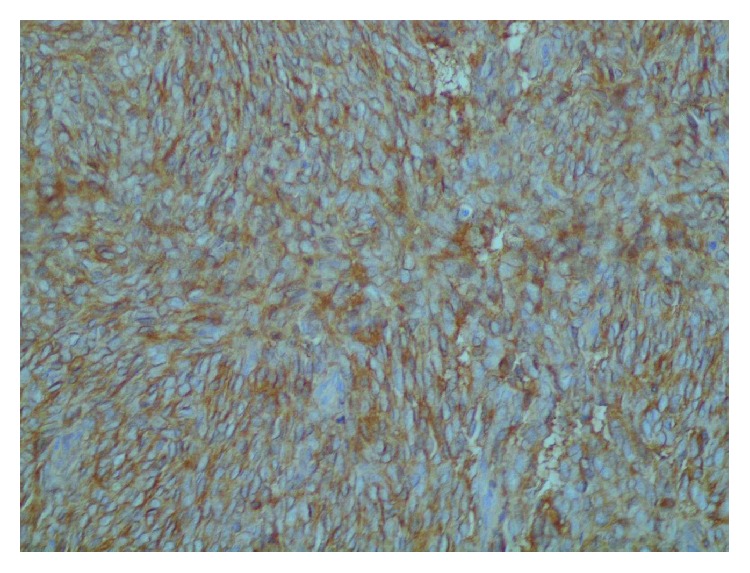
CD10-positive staining for the left mass suggesting stromal origin.

Surprisingly, histopathology of excisional biopsy performed to the left breast mass showed high cellularity of spindle-shaped cells; the mass contained fatty tissue and showed an abundant mitotic activity (Figure 4). Immunohistochemistry (IHC) showed negative results for epithelial markers, such as cytokeratin 7 (CK7) (Figure 5) and epithelial membrane antigen (EMA), leukocyte common antigen (LCA), and desmin, which in turn excluded carcinoma origin, lymphoma/leukaemia, and muscular origin, respectively. Positive staining of CD10 (Figure 6) confirmed stromal origin confirming the diagnosis of high-grade stromal sarcoma of the breast.

Repeating CT scan and scintigraphy showed no signs of metastatic disease.

Consequently, she underwent bilateral mastectomy and bilateral axillary lymph node resection. Histopathology showed free surgical margins and no invaded nodes.

In the follow-up, the patient had received hormonal therapy (Tamoxafen) after adjuvant chemotherapy: 8 cycles of Taxol 150 mg+Cisplatin 50 mg and 3 sessions of radiotherapy. The follow-up for 14 months showed no evidence of recurrence.

## 3. Discussion

This is the first case to be reported with concomitant contralateral breast stromal sarcoma and carcinoma from Syria. When bilateral breast cancer is present, it is usually the same type for both breasts [7].

Reviewing the literature (PubMed and Google Scholar search, March 2019), we found no identical cases of coexisting primary stromal sarcoma and invasive ductal carcinoma in distinct breasts. However, few similar cases reported bilaterally different types of cancer with sarcoma. In de Mello et al.'s report [8], a 42-year-old woman had a lobular pleomorphic carcinoma in the right breast, a different type of carcinoma compared to that in our case, and a sarcoma in the left that was diagnosed histologically.

On one hand, primary sarcomas can take various histological types that often require IHC to differentiate [9], and secondary sarcomas often present as angiosarcoma, especially after radiation therapy of another tumor [10]. In our patient, it was important to do IHC to confirm the diagnosis as it is uncommon to be considered a different diagnosis in a previous carcinoma patient who never received radiotherapy.

On the other hand, the mainstay treatment in soft tissue sarcoma is surgery [4, 11, 12]. Axillary resection is not necessary, since sarcomas rarely invade the lymphatic system [3]. We followed these management protocols of surgery. However, axillary nodes were dissected due to the presence of concomitant carcinoma.

Also, in high-risk cases, adjuvant and neoadjuvant chemotherapy and radiotherapy should be considered and chemotherapy is the mainstay of treating widespread metastatic cancer, whereas radiotherapy is preferred for lymphatic metastasis and reducing the rate of locoregional recurrence [13].

The first cancer was diagnosed early in the disease course giving a good recovery chance. After presenting again with bilateral masses, the case necessitated a more radical surgery with adjuvant chemotherapy, radiotherapy, and hormonal therapy. In response to this management plan, she had a disease-free state for 14 months.

## 4. Conclusion

This case presents a rare entity of bilaterally different types of cancers including stromal breast sarcoma. This report highlights the importance of a profound study of new lesions previously diagnosed as breast cancer lesions to not miss the diagnosis of different types of cancer and thus be wrongly treated. This is the first case from Syria to be reported.

## References

[1] Trent J. C., Benjamin R. S., Valero V. (2001). Primary soft tissue sarcoma of the breast. *Current Treatment Options in Oncology*.

[2] Berg J. W., Decrosse J. J., Fracchia A. A., Farrow J. (1962). Stromal sarcomas of the breast. A unified approach to connective tissue sarcomas other than cystosarcoma phyllodes. *Cancer*.

[3] Cil T., Altintas A., Pasa S., Buyukbayram H., Isikdogan A. (2008). Primary spindle cell sarcoma of the breast. *Breast Care*.

[4] Chen Y., Thompson W., Semenciw R., Mao Y. (1999). Epidemiology of contralateral breast cancer. *Cancer Epidemiology and Prevention Biomarkers*.

[5] Balleyguier C., Ayadi S., Van Nguyen K., Vanel D., Dromain C., Sigal R. (2007). BIRADS classification in mammography. *European Journal of Radiology*.

[6] Gradishar W. J., Anderson B. O., Balassanian R. (2018). Breast cancer, version 4.2017, NCCN Clinical Practice Guidelines in Oncology. *Journal of the National Comprehensive Cancer Network*.

[7] El Hanchi Z., Berrada R., Fadli A. (2004). Cancer du sein bilatéral. Incidence et facteurs de risque. *Gynécologie Obstétrique & Fertilité*.

[8] de Mello R. A., Figueiredo P., Marques M., Sousa G., Carvalho T., Gervásio H. (2010). Concurrent breast stroma sarcoma and breast carcinoma: a case report. *Journal of Medical Case Reports*.

[9] Takahashi H., Inaba S., Yabuki H. (2016). Two cases of stromal sarcoma of the breast. *Gan to kagaku ryoho. Cancer & chemotherapy*.

[10] Li N., Cusidó M. T., Navarro B. (2016). Breast sarcoma. A case report and review of literature. *International Journal of Surgery Case Reports*.

[11] Chuba P. J., Hamre M. R., Yap J. (2005). Bilateral risk for subsequent breast cancer after lobular carcinoma-in-situ: analysis of surveillance, epidemiology, and end results data. *Journal of Clinical Oncology: Official Journal of the American Society of Clinical Oncology*.

[12] Skowronek J., Piotrowski T. (2002). Bilateral breast cancer. *Neoplasma*.

[13] Lum Y. W., Jacobs L. (2008). Primary breast sarcoma. *The Surgical Clinics of North America*.

